# Assessing the Outputs, Outcomes, and Impacts of Science Communication: A Quantitative Content Analysis of 128 Science Communication Projects

**DOI:** 10.1177/10755470241253858

**Published:** 2024-06-17

**Authors:** Sophia Charlotte Volk

**Affiliations:** 1 University of Zurich, Switzerland

**Keywords:** evaluation, impact measurement, content analysis, mass media, public engagement, science communication training, science outreach, social media, science policy

## Abstract

Few studies have explored how science communication projects are evaluated and what impact they have. This study aims to fill this gap by analyzing the results of science communication projects carried out by academics. Drawing on the theory of change and evaluation models, possible results of science communication projects are conceptually distinguished at the levels of outputs, outcomes, and impacts. The study draws on a dataset of 128 science communication projects funded by the Swiss National Science Foundation from 2012 to 2022. Quantitative content analysis reveals few rigorous evaluation designs and a focus on reporting outputs, while outcomes and societal impacts are often neglected.

## Introduction

Recently, there has been an increasing interest in measuring the impact of science communication in both research and practice (Jensen, 2014; Weitkamp, 2015). As science communication continues to play an essential role at the interface between science and society, understanding its effects and societal impact becomes more important for policymakers, researchers, and science communicators alike (Fecher & Hebing, 2021; Fu et al., 2016). Science communication is often associated with the goal of stimulating interest in science, promoting public engagement, or improving scientific literacy (Metcalfe, 2019; Weingart & Joubert, 2019). However, it can also have unintended and unexpected negative consequences, such as diminishing interest or trust in science (Watermeyer, 2012). Therefore, it is important to empirically examine whether science communication activities achieve their intended effects in practice or not (Jensen, 2019).

As governments around the world come under growing pressure to demonstrate that investments of public money in research have positive impacts on society, funding agencies increasingly expect academics to engage in science communication with the public and sometimes allocate their own budgets for this purpose (Hill, 2016; Palmer & Schibeci, 2014). Along with this, expectations for evaluation of public outreach and engagement are increasing (Fogg-Rogers et al., 2015). Although the importance of evaluating and measuring the effects of science communication has been emphasized, it is often neglected in practice (Jensen, 2019). Large-scale assessments of science communication projects based on project evaluations are relatively rare (for exceptions see, e.g., Fu et al., 2016; Ziegler et al., 2021), especially with regard to outreach activities carried out by academics.

This study aims to fill this gap by examining how academics evaluate their own science communication projects, if at all, and what exactly the effects of their projects are. Drawing on the theory of change and the idea of logic models, the effects of science communication are distinguished at the levels of outputs, outcomes, and impacts. Empirically, this study relies on a unique dataset of all 128 science communication projects funded by the Swiss National Science Foundation (SNSF) between 2012 and 2022. For a comprehensive meta-analysis of these projects, the SNSF granted access to academics’ project applications, final reports, and project data. A standardized quantitative content analysis was conducted to uncover gaps in evaluation practices and between promised and realized results.

## Literature Review

The increasing importance of communicating about science and engaging the public has led funding agencies to invest significantly in science communication programs (Hill, 2016). More and more government funding agencies are requiring academics to develop an outreach plan for communicating with lay audiences in addition to conducting excellent research (Brenninkmeijer, 2022; Palmer & Schibeci, 2014). As a result, academics are increasingly incentivized to communicate their research, for example, appearing as experts in mass media or at public forums (Peters, 2021; Scheu et al., 2014). However, relatively few of them are trained in science communication (Ho et al., 2020; Rauchfleisch et al., 2021). As the science communication funding landscape expands and budgets rise, so do funders’ expectations for demonstrating tangible impacts and accumulating evidence of effective science communication practices (Fogg-Rogers et al., 2015; Jensen & Gerber, 2020; King et al., 2015). One of the driving countries is the United Kingdom, which introduced a standardized evaluation framework, the Research Excellence Framework (REF), in 2014 to guide a nation-wide assessment of the societal impact of research (Hill, 2016). The San Francisco Declaration on Research Assessment (DORA), launched a decade ago, has stimulated numerous efforts around the globe to improve evaluation and measure the real-world impact of research on society (Gagliardi et al., 2023). As a result, policy makers, institutions, and funding agencies in various countries—including the Netherlands, Sweden, Italy, Australia, and the United States—require academics to assess the impact of their research not only in scientific terms but also in terms of societal impact (Fogg-Rogers et al., 2015; Jensen et al., 2022).

### Academics as Science Communicators

Given the important role of individual academics in the science-society nexus, there is a growing body of research on academics as science communicators (Calice et al., 2023). Numerous studies have investigated academics’ motivations for communicating with lay audiences (e.g., Kessler et al., 2022; Rauchfleisch et al., 2021) or their understanding of public engagement (e.g., Davies, 2013; Watermeyer, 2012). Other studies have explored the need for and effectiveness of science communication trainings for academics (e.g., Ho et al., 2020; Rodgers et al., 2018). Research shows that academics have a rather broad understanding of public engagement (Davies, 2013; Watermeyer, 2012), ranging from “one-way” activities that focus on information sharing and education such as public lectures, representing a knowledge-deficit mind-set (e.g., Besley & Nisbet, 2013; Metcalfe, 2019), to “two-way” activities such as deliberative forums, open days, or science festivals (Calice et al., 2023; Ho et al., 2020). However, little is known about how academics subsequently self-evaluate the results and report on the impact of their outreach formats.

In the field of research evaluation, a distinction is often made between two kinds of impact: *societal* impact and *scientific* impact (D’Este et al., 2018). In the broadest sense, impact is understood as the long-term, substantial result or value of research. An extensive literature review of the term “societal impact” of research shows that different labels and different dimensions of impact are discussed (Bornmann, 2013), including impact on society, education, practice, economy, public health, or the environment (see also Jensen et al., 2022). Studies examining how academics understand the societal impact of their research show that impact goals differ depending on the discipline: While the humanities and social sciences often emphasize educational impact, the natural sciences tend to focus more on environmental, technological, or health impacts (Fecher & Hebing, 2021). Empirical impact assessment is often difficult and based on narrative impact stories or case studies with stakeholders (e.g., Jensen et al., 2022), rather than quantifiable or monetary values (e.g., for the economy). In contrast, indicators of *scientific* impact are often measured quantitatively, including publication or citation metrics (e.g., h-index and impact factor of journals), awards, or acquisition of follow-up grants (Gagliardi et al., 2023).

Since funders increasingly require academics to assess and report both on the scientific and societal impact of their research (Brenninkmeijer, 2022), there is growing criticism that funders impose unrealistic expectations for societal impact, which may lead academics to “overclaim” (potential) impact in order to improve their chances of success in the fierce competition for third-party funding (King et al., 2015). This goes hand in hand with a development that some refer to as impact “inflation” or “utopianism” in grant applications (Chubb & Watermeyer, 2017) driven by increased pressures on academics to “marketize” impact. When it comes to reporting on the impact achieved, there is also a growing risk that self-reported evaluations of research—especially when disclosed to funders—may degenerate into a mere “success story,” while unachieved results are omitted or glossed over (King et al., 2015; Ziegler et al., 2021).

### Evaluation of Science Communication

There is a long tradition in science communication research of studying the short- or medium-term effects of science communication messages, formats, or visuals on audiences (Besley et al., 2018), demonstrating a breadth of cognitive, attitudinal, emotional, behavioral, or physiological changes (e.g., König et al., 2023; Mede, 2022). Effects studies are often based on experiments or experimental surveys and attempt to isolate the effects of science communication on audiences (Mede, 2022), which often has the disadvantage of low external validity and generalizability to other contexts. In real-world science communication scenarios, the use of such controlled study designs is often not feasible. For assessing science communication in field settings, *evaluation* comes into play, an area that is receiving increasing research interest (e.g., Jensen & Gerber, 2020; Niemann et al., 2023). Evaluation is concerned with systematically assessing the value of an object—in this case, science communication. A basic prerequisite of evaluation is the definition of goals at the beginning of a science communication project, on the basis of which the results achieved can be assessed (Raupp & Osterheider, 2019). This assessment requires measurement, i.e., an empirical process that makes use of quantitative and qualitative research methods (Pellegrini, 2021). The measurement results obtained serve as indicators (“metrics”) to compare the intended goals with the actual results, allowing conclusions to be drawn about goal achievement and the effectiveness of science communication (Volk, 2023). In this way, evaluation serves learning and continuous improvement, as well as accountability to third parties such as funders.

Empirical studies regarding science communication evaluations in practice are scarce. Three types of studies can be found: First, *case studies* on specific formats such as science festivals (Adhikari et al., 2019; Boyette & Ramsey, 2019; Pennisi & Lackey, 2018), science slams (Niemann et al., 2020), citizen science (Andersen et al., 2021), school interventions (Ruiz-Mallén et al., 2018), or workshops for teachers (King et al., 2015). Such studies are often based on *external* evaluations by researchers (as scientific accompanying research) and rely on surveys or interviews that explore audiences’ motivations to participate in an activity, their attitudes toward science, knowledge gains, or behavioral intentions. Second, *survey* or *interview studies* with professional science communicators working for universities (e.g., Bühler et al., 2007; Höhn, 2011; Sörensen et al., 2024), scientific institutions (e.g., Metcalfe et al., 2012), or for citizen science projects (e.g., Phillips et al., 2018), who self-report about their *self*-evaluation practices. Third, *content and meta-analyses* of public or non-public (self-)evaluation reports of science communication projects (e.g., Fu et al., 2016; Ziegler et al., 2021) or of published impact case studies (e.g., Jensen et al., 2022), which provide insights into evaluation methods and designs.

Two common findings stand out in these different studies: First, evaluation of science communication is considered important, but still not widespread, and is often based on self-reports (see Jensen, 2014; Phillips et al., 2018; Watermeyer, 2012). Typically, evaluations focus on easily countable indicators such as media coverage or visitor numbers (e.g., Bühler et al., 2007; Höhn, 2011; Weingart & Joubert, 2019). The emphasis on media attention is partly driven by funding bodies that attach importance to mass media coverage (Scheu et al., 2014; Weingart, 2022). Measurement of effects is often not robust, as sophisticated designs capable of capturing changes are rare (Ziegler et al., 2021). Second, the hoped-for effects on participants’ attitudes or behavior are often small, possibly indicating that the intended goals were too ambitious (Pellegrini, 2021).

### Evaluation Stages

Since evaluations aim to attribute effects to specific science communication projects or interventions, they can draw on “logic models” that distinguish different stages of effects and make assumptions about logical pathways between the stages to achieve these effects (Friedman, 2008). Such models reflect assumptions from program theory and the theory of change (Clark & Taplin, 2012; Funnell & Rogers, 2011; see also Macnamara, 2018), which posit that a specific project or intervention will lead to certain anticipated and desired changes through a series of stages, at each of which data can be collected to measure change. This idea, widely used in public administration and research evaluation, has also proven fruitful for the evaluation of science communication, as it helps to disentangle and measure the communication effects of outreach activities at different stages (Friedman, 2008; Fu et al., 2016; Weitkamp, 2015).

Compared with evaluation in other fields, the evaluation of communication is arguably challenged by fundamental difficulties in capturing “change”: Measuring communication effects is difficult and scholars are often unable to establish direct effects between exposure to communication and cognitive, attitudinal, or behavioral changes (Brinberg & Lydon-Staley, 2023). This is because communication or media effects are often indirect, conditional, transactional and person-specific, and may occur with a delay (Valkenburg et al., 2016); hence, it is difficult to attribute causality to exposure to communication (Mede, 2022). Compared with other subfields of communication or advertising, science communication is moreover special because it is characterized by the uncertainty and complexity inherent in science-related topics and is often directed toward a general public rather than a specific target group (e.g., Metag, 2017). Studies show that individuals differ strongly in their predispositions toward science (e.g., Schäfer et al., 2018) and that such predispositions are quite stable (Metag, 2017) and rather difficult to change through communication. Overall, these characteristics make evaluation of science communication and measurement of changes per se challenging. This is exacerbated when the responsibility for the evaluation of a specific temporary science communication project lies with academics, who are not specifically trained in science communication or its evaluation and may not be familiar with evaluation models, methods, and metrics.

**Figure 1.**
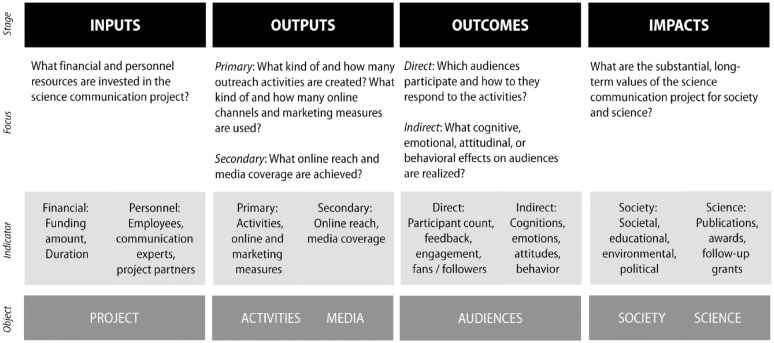
Conceptual model for evaluation of science communication projects.

At their core, science communication evaluation models distinguish between four stages (Raupp & Osterheider, 2019; Volk, 2023; similarly Pellegrini, 2021): *inputs, outputs, outcomes*, and *impacts*. Between these stages, logical ordered and causal relationships are assumed to exist, that is, it is assumed that outputs logically lead to certain outcomes, which in turn are expected to lead to certain impacts. Adapting this conceptualization from the literature, a model is proposed in Figure 1 to guide empirical evaluations of science communication projects along the four stages.

For each stage, the model presents the focus of evaluation and illustrates possible indicators and the object of evaluation (inspired by DPRG & ICV, 2011):

- *Inputs* comprise the resources invested in science communication over time and are measured in relation to the project. Adequate financial and personnel resources are crucial for creating, organizing, promoting, and evaluating outreach activities (Cooke et al., 2017). This can also include the network of collaborations with partners such as science museums, educational institutions, or media outlets, or the support of internal communication experts (Besley et al., 2021; Cooke et al., 2017).
- *Outputs* can be distinguished into internally generated *primary* outputs (also: “activities”) and externally achieved *secondary* outputs. *Primary outputs* include the type and number of outreach activities created to inform, educate, or engage audiences (e.g., exhibitions or science cafés), as well as the type and number of online communication (e.g., social media) and marketing channels (e.g., media relations or advertisements) used to promote these activities (Van Dyke & Lee, 2020; Weingart & Joubert, 2019). *Secondary outputs* include the media coverage and online reach achieved by science communication projects, both of which are widely used indicators of success (Cooke et al., 2017; Raupp & Osterheider, 2019). Reach shows, for example, how many people have been exposed to the content created (e.g., by tracking impressions and visits) (Phillips et al., 2018; Volk, 2023), and is the prerequisite for the realization of effects at the outcomes stage.
- *Outcomes* include the effects of science communication on the audience and can be divided into *direct outcomes* (usually short-term) and *indirect outcomes* (usually medium-term). *Direct outcomes* include the number of people participating in outreach activities and their immediate feedback or response. The latter can be measured by tracking social media engagement (e.g., likes and shares), analyzing sentiments in user comments, or analyzing the growth of followers, fans, or subscribers (Raupp & Osterheider, 2019; Volk & Buhmann, 2023). Counting visitors is one of the most commonly used quantitative indicators (Phillips et al., 2018; Weingart & Joubert, 2019), although a more meaningful indicator is, for example, whether participants have developed a greater interest in or enjoyed being exposed to an activity. Such *indirect outcomes* are about the medium-term effects of science communication: Participation in outreach activities can lead to *changes* in people’s *cognitions* (e.g., knowledge and understanding), *emotions* (e.g., enthusiasm and fascination), *attitudes* (e.g., opinion change), or *behavior* (e.g., recommendation and repeat participation) (Mede, 2022; Phillips et al., 2018). Compared with direct outcomes, measuring indirect outcomes requires more complex evaluation designs and research methods.
- *Impacts* include the long-term, substantial benefits or values of science communication projects that extend beyond the participants. Measuring *societal impact* is complex because impacts are often delayed and contingent, and it is difficult to attribute causality to science communication (Fecher & Hebing, 2021; King et al., 2015). It can be demonstrated in narrative statements or impact case studies that illustrate achieved benefits for society (e.g., trust in science), education (e.g., scientifically educated workforce), the environment (e.g., acceptance of technological innovations), the economy (e.g., patents), or other areas (Bornmann, 2013). For measuring *scientific impact*, various established quantitative indicators can be used, such as publication or citation metrics, awards, or follow-up grants (Gagliardi et al., 2023; Jensen et al., 2022).

### Evaluation Methods and Designs

Sound evaluation methods and designs are essential to record the effects of science communication projects along the above-mentioned stages (Jensen, 2019). The entire spectrum of qualitative and quantitative social science research methods can be used, including standardized surveys, semi-structured interviews or focus groups, observations of participants, experiments, eye tracking or knowledge tests (e.g., Fu et al., 2016; Niemann et al., 2023; Pellegrini, 2021). Especially for evaluating interactive activities such as science festivals, a number of autonomous and informal feedback methods can be used, such as graffiti walls, snapshot interviews, before-and-after-drawings, guestbooks or feedback cards (Campos, 2022; Grand & Sardo, 2017). Moreover, content analyses of media resonance, website or social media analyses can be used to evaluate mediated formats of science communication. Different evaluation methods are typically combined in multimethod designs to measure science communication projects along different stages (Grand & Sardo, 2017).

Evaluation can take place before the outreach activity (“formative evaluation”), during the activity (“processual evaluation”), after the activity (“summative evaluation”), or both before and after (pre- and post-test-design) (Fu et al., 2016; Grand & Sardo, 2017). To reliably measure knowledge or attitude changes among participants, pre- and post-test-designs and a comparison of the measurement results before and after the activity are inevitably required. In practice, however, evaluation is more often carried out “summatively” (Ziegler et al., 2021), that is, visitors self-report whether they have learned something after participating in an activity and have to assess their knowledge gain themselves. Control groups are very rarely used in science communication evaluations to assess whether there are differences between groups that have (not) been exposed to an outreach activity (Pellegrini, 2021). The fact that elaborate evaluation designs are still rare in practice is often attributed to a lack of time and budget or missing knowledge on the part of the evaluators (Grand & Sardo, 2017; Jensen, 2019; Pellegrini, 2021). In addition, lack of support and interest in evaluations as well as concerns about possible negative results are also barriers (Sörensen et al., 2024; Volk, 2023).

While there is no large-scale empirical evidence on the uptake of evaluation models for science communication, there are indications that several funding agencies (e.g., the Commonwealth Scientific and Industrial Research Organization (CSIRO) in Australia or the Science Foundation Ireland (SFI) in Ireland) as well as some university communication departments (Sörensen et al., 2024) have adopted the idea of logic models for science communication evaluation. However, it remains unclear how academics engaging in science communication projects evaluate their own efforts, if at all, and whether they draw on such models, and what methods they adopt. Moreover, it is not known what evidence is created by such evaluations about the effects of science communication at different stages. Thus, two research questions (RQs) were formulated:

- *RQ1*: How are science communication projects evaluated, if at all, and what methods are used for this purpose?
- *RQ2*: What are the outputs, outcomes and impacts of the science communication projects?

## Data and Method

To answer the research questions, this study analyzed a dataset comprising a full population of 128 science communication projects funded by the SNSF over the last 10 years. Switzerland has a competitive higher education system with world-renowned universities, and Swiss academics are quite active in the field of science communication (Rauchfleisch et al., 2021). Since 2012, the SNSF has been funding science communication projects that promote dialog between science and society with up to 200,000 Swiss Francs (CHF) for a maximum of 36 months as part of the “Agora” funding program. The Agora scheme is open to all researchers holding a Ph.D. at Swiss institutions in all disciplines, regardless of their experiences in science communication. On the recommendation of the SNSF, the projects often involve not only researchers but also communication experts and external partners who have the expertise (e.g., pedagogical and artistic qualifications) or networks (e.g., with associations) necessary for the successful implementation of outreach projects. Funding applications are reviewed by external reviewers and funding decisions are made by a commission of academics and professional science communicators.

### Dataset

The SNSF made available data from its archives for all 128 science communication projects funded since 2012 and concluded by May 2022. Access to non-public data archives of funding organizations is rare, and although ex-post assessments of funding lines are often commissioned externally by funders, the results of such assessments are rarely published (Bornmann et al., 2007), so there are few studies examining patterns in funding schemes (for exceptions, see, e.g., van der Lee & Ellemers, 2015; Witteman et al., 2019). The SNSF provided the author with access to three document sources for each project: (a) *grant applications*, (b) *final project reports*, and (c) *project data*. While the latter contained selected project results in a standardized format in Excel, the former documents included academics’ own descriptions of the planned and realized project goals, outreach activities, the evaluation concept, and the outputs, outcomes, and impacts achieved. The analysis of (a) grant applications and (b) final project reports enabled comparisons to be made between promised and achieved results.

Of course, several limitations must be considered when interpreting such data: It can be assumed that academics have an interest in presenting their projects as impactful or successful to funders (King et al., 2015). As the analysis is based on self-reports by the grant holders and not an independent assessment by the external reviewer committee, it is not possible to ascertain whether the reported results are valid, actually correct, or presented in an exaggeratedly positive manner. Nonetheless, the dataset is an interesting source for analyzing whether or not robust self-evaluations occur, what metrics academics use to signal success, whether they also report failures, and whether there are gaps between promised and realized results.

A complete sample description of the 128 science communication projects can be found in Supplementary Material 1. All data protection and privacy regulations for handling confidential data and project information were adhered to (see Supplementary Material 2). The total page count for each project averaged 40.4 pages (*SD =* 45.3). In sum, the material comprised 5,174 pages.^1^ Quantitative content analysis was used to analyze the full texts of all three sources.

### Operationalization and Data Analysis

Data analysis took place from July to October 2022. Three student coders were responsible for coding the material after attending an intensive 2-week coding training led by the author, which covered roughly 20% of the material and in which coding inconsistencies were intensively discussed and jointly resolved. The material was provided in English, but due to Switzerland’s multilingual characteristics, a few sources were obtained in French, German, or Italian, which were then coded by the coders with bilingual backgrounds. The entire material for each project, including the (a) *grant applications*, (b) *final project reports*, and (c) *project data*, was double-coded by two coders. The codebook comprised 76 variables and was theoretically derived from the literature and the distinction between the evaluation stages in the conceptual model (Figure 1). The relevant variables are described below (see also Supplementary Material 3). For most variables, both the planned results in the grant applications and the achieved results in the final project reports were coded, using the same operationalization to allow comparisons.

Overall intercoder reliability was satisfactory given the extensive length of the project descriptions and the high complexity of the material, with an average percent agreement rate of 82% between two coders. All inconsistencies between the double codings were checked and resolved by the author by reviewing the original material in a final step and deciding on the definitive coding to ensure a high standard of analytical quality.

#### Evaluation Methods and Designs

To answer RQ1, it was coded whether an evaluation was conducted (agreement: 81%), and what type (agreement: 78%; e.g., summative), what design (agreement: 64%; e.g., qualitative), and what methods (agreement: 89%; e.g., feedback and survey) were used (following, e.g., Fu et al., 2016; Grand & Sardo, 2017; Pellegrini, 2021; Phillips et al., 2018; Ziegler et al., 2021).

#### Outputs, Outcomes, and Impacts

To answer RQ2, the planned and achieved outputs, outcomes, and impacts of the science communication projects were coded.

For *primary outputs*, the number and type of activities were recorded, using a list of possible activities (agreement: 81%; inspired by, e.g., Fähnrich, 2017; Fu et al., 2016). Moreover, the type of online channels (agreement: 92%; e.g., website) and marketing measures (agreement: 83%; e.g., TV ads) were coded.

*Secondary outputs* were operationalized as media coverage and online reach (Raupp & Osterheider, 2019): For media coverage, the number of reports in TV, radio, print, and online news and on digital media was open-coded numerically (agreement: 73%) based on the SNSF (c) *project data* form and subsequently aggregated.^2^ In addition, it was coded whether media tonality was mentioned in the final reports and described as positive, neutral, or negative (agreement: 93%). Finally, it was coded whether online reach was measured (agreement: 86%), and when it was, the number of visits or visitors for websites and reach or impressions for each social media channel were recorded (e.g., Volk, 2023).

For *direct outcomes*, it was coded whether social media engagement (agreement: 86%; e.g., likes and shares), sentiments (agreement: 96%; e.g., positive, mixed, negative), and follower or fan growth were reported (agreement: 95%) (Raupp & Osterheider, 2019), and the exact metrics were then open-coded. Participant count was open-coded numerically (agreement: 80%) and aggregated at the project level when numbers were distinguished for different activities (offline, online).^3^ Moreover, the main type of participants was coded (agreement: 73%; e.g., school students), and when reported, participant feedback was coded as positive, mixed, or negative (agreement: 82%) (Weingart & Joubert, 2019).

For *indirect outcomes*, it was coded whether and which effects on participants (agreement: 84%; e.g., cognitive and emotional) were reported (e.g., Mede, 2022), and whether effect statements were empirically founded or not (agreement: 68%).

The type of *societal impact* reported was coded (agreement: 95%), informed by previous differentiations (Bornmann, 2013; Fecher & Hebing, 2021). Only actual long-term and substantial effects were coded as impacts, to counteract the problem that the term “impact” may be used inflationary, sometimes even artificially, or as a synonym for any kind of effect (Chubb & Watermeyer, 2017). *Scientific impacts* were open-coded numerically given that academics reported the number of publications^4^ (agreement: 72%), awards (agreement: 97%), and follow-up projects (agreement: 94%) in the (c) *project data* form in a standardized format (Gagliardi et al., 2023), which academics could update after project completion.

The data analysis relied on descriptive statistics and was carried out in SPSS.

## Results

The analysis of the 128 science communication projects shows how such projects are evaluated, if at all, and with what methods (RQ1), and what results these projects have achieved in terms of outputs, outcomes, and impacts (RQ2). As expected, almost all projects were described by grantees as successful in achieving their goals. But quite often, these were unsubstantiated claims: Almost one-third of projects had not conducted evaluations, and the outputs, outcomes, and impacts achieved were often not transparently reported.

### Evaluation Methods and Designs

The results for RQ1 show that 68.7% of the 128 projects conducted an evaluation, while 31.3% did not (see Table 1). There are few mixed methods approaches and rigorous evaluation designs: 43% chose a single method for evaluation, 19.5% combined two evaluation methods, and 8 projects triangulated three or more evaluation methods. The most frequently used methods were feedback methods (42.2%) and standardized surveys (35.2%), followed by user research (7.8%; e.g., analysis of user data collected via citizen science apps). The share of projects using a mainly qualitative (28.9%) or mainly quantitative evaluation design (25.8%) was almost equal; a combination of qualitative and quantitative methods was found in 13.3% of projects. In the remaining projects, the design remained unclear. Summative evaluation at the end of a project was the most common (53.1%). Pre- and post-test-designs relying on before-and-after measurements were used in 8.6% of projects—however, this would be precisely necessary to reliably record changes in audiences’ knowledge, attitudes, or behavioral intentions. In 7% of projects, a processual evaluation was reported.

**Table 1.** Evaluation Type, Design, and Methods of Science Communication Projects (*N* = 128).

Item	Operationalization	%
Evaluation	Reported	68.7
	Not reported	31.3
Type of evaluation	Summative (ex-post)	53.1
	Pre- and post-test-design	8.6
	Processual evaluation (continuous)	7.0
	Formative (ex-ante)	0
	Not applicable	31.3
Evaluation design	Mainly qualitative (semi-standardized)	28.9
	Mainly quantitative (standardized)	25.8
	Mixed (qualitative and quantitative)	13.3
	Unclear/not applicable	32.0
Evaluation methods^a^	Feedback methods (e.g., guestbook)	42.2
	Standardized surveys	35.2
	User research (e.g., of data collected through apps)	7.8
	Observations	6.3
	Knowledge tests	5.5
	Semi-structured interviews	5.5
	Experiments	0
	Other	0.8
	Unclear/not applicable	31.3

a Multiple answers were possible.

In most projects, it was not identifiable that a specific evaluation model was used for the purpose of evaluation. Only in a handful of projects did academics explicitly base their evaluation on a logic model that distinguished outputs, outcomes, and impacts and described the logical pathways leading to intended results.

### Outputs, Outcomes, and Impacts of Science Communication Projects

The findings for RQ2 reveal what kind of activities were created in the science communication projects and how academics report on the results of their projects.

#### Outputs

For *primary outputs*, the 128 projects created a bundle of activities and used various communication measures to reach their target audiences (see Table 2). For activities, a total of 33 different formats were identified, and on average, M = 4.45 activities were carried out per project. One *main* activity was identified for each project: Exhibitions or installations (39.8%) were clearly most common, followed by workshops or lectures (18.0%) and online platforms or blogs (10.2%). Although these formats represent rather traditional, unidirectional approaches, the majority of projects still offered possibilities for participation and interaction with researchers (e.g., through games or live experiments).

**Table 2.** Primary Outputs of Science Communication Projects (*N* = 128).

Item	Operationalization	%
ACTIVITIES		
Main activities^a^	Exhibition, installation	39.8
	Workshop, lectures	18.0
	Online platform	10.2
	Learning/teaching material	7.0
	App	4.7
	Science performance, show	4.7
	Film, video, movie	3.1
	Other (e.g., science festival, MOOC, podcast, game)	12.8
COMMUNICATION		
Online communication channels^a^	Website	82.0
	Facebook	35.9
	YouTube	17.2
	Twitter/X	16.4
	Instagram	7.0
	Other/unspecified social media channel	16.4
	Not reported	12.5
Marketing measures^a^	Promotion through network of partners	57.8
	Public poster, flyer, billboard	43.8
	Media relations	42.2
	Newsletter, direct mailing	38.3
	Advertisement (e.g., TV and radio)	13.3
	Not reported	23.4

a Multiple answers were possible.

For online communication, most projects used a website (82.0%), whereas social media use was comparatively low, led by Facebook (35.9%), YouTube (17.2%), and Twitter/X (16.4%). Overall, 61.7% used more than one online channel. 12.5% did not report using online media to address their target audiences. To increase public visibility, projects also used traditional marketing measures: They most often relied on partners to promote outreach activities through their network (57.8%), created posters or flyers (43.8%), used media relations (42.2%), or sent out direct mailings or newsletters to reach target groups (e.g., teachers) (38.3%).

Notably, one-third of the projects (31%) reported that they were not able to successfully carry out all the planned activities, but that they had to deviate from their plans to develop or implement (a certain number of) activities due to various obstacles. In these cases, detailed justifications were given in the final reports for not fulfilling certain project goals set out in the grant application. Most of the obstacles were related to COVID-19 pandemic constraints, but a few also mentioned difficulties in the project team, unrealistic goal setting, or unforeseen problems with project planning and staffing.

For *secondary outputs*, the 128 projects focused on reporting the volume of media coverage, while neglecting reporting online reach (see Table 3). Overall, the projects generated considerable media interest: In absolute terms, they resulted in 1,437 media reports, which equals an average of M = 11.2 media appearances (MDN = 5) per project, either on radio or TV, in print and online news, or on digital media (e.g., blogs), mainly in German- and French-speaking Swiss media. Media coverage varied depending on discipline and activity type.^5^ At least 24.2% of projects did not report on any media attention. Notably, 12% of projects indicated not only the amount of coverage in the media but also provided a qualitative indication that media reports were positive, although this was never based on a media response analysis.

**Table 3.** Secondary Outputs of Science Communication Projects (*N* = 128).

Item	Operationalization	%
Coverage in the media	More than 30 reports	11.1
	20 to 29 reports	7.2
	10 to 19 reports	13.2
	1 to 9 reports	44.6
	Not reported	24.2
Reach of online channels	Reported	38.3
	Not reported	49.2
	Not applicable	12.5

In terms of online communication, while most projects used some type of online channel, only 38.3% reported whether people had possibly seen the content. Online reach was most commonly reported for websites (30.4%). However, the metrics and time periods reported varied widely, making it impossible to compare the data and accumulating evidence of effective science communication online.^6^

#### Outcomes

For *direct outcomes*, the results reveal that the projects emphasize participant numbers and feedback, with fewer reporting on immediate reactions or responses to online content (see Table 4). Overall, the projects attracted a considerable number of participants, totaling more than half a million participants (excluding one lighthouse project that attracted 500,000 visitors): On average, they reached M = 6,433 people (*SD* = 11,176; MDN = 1,400), but visitor count varied greatly depending on the type of activity,^7^ and few projects reported whether participants were just by-passers or actually engaged with the activities. For 22.7% of projects, the exact number of people reached remained unclear. Most grantees reported that the target audiences were reached as planned (77%), but at least 8% self-critically indicated that the project had not successfully reached certain target groups such as socio-economically disadvantages children. The remaining 15% of projects did not specify whether the activities had reached the predefined target groups. While most projects indicated which target audiences were ultimately reached, with the most common main target group being the general public (45.3%), followed by students or young people (39.1%), only 21% provided detailed information on the demographics of participants (e.g., age, gender, or interest in science). Four of five projects addressed an audience already interested in science rather than an audience distant to science. For the remaining projects, the question of who exactly the participants were in terms of socio-demographics remained vague at best. Most projects provided an estimation of the feedback of participants (73%), which was often positive (68%); 5% self-critically admitted that the projects had also encountered negative or mixed feedback. 27% of projects did not report on the initial reactions of participants. In 33.6% of projects, empirical evidence for the feedback was provided (e.g., obtained through a feedback form or visitor survey), while 33.6% remained vague on how participant feedback was obtained, and no information was found in the remaining reports.

**Table 4.** Direct and Indirect Outcomes of Science Communication Projects (*N* = 128).

Item	Operationalization	%
DIRECT OUTCOMES		
Social media engagement	Reported (e.g., likes and shares)	21.9
	Not reported	41.4
	Not applicable	36.7
Social media sentiments	Reported (e.g., positive tonality)	2.4
	Not reported	54.7
	Not applicable	43.0
Follower or fan growth	Reported	12.5
	Not reported	43.0
	Not applicable	44.5
Number of participants	More than 100,000 participants	0.8
	30,000 to 99,999 participants	3.9
	10,000 to 29,999 participants	10.9
	3,000 to 9,999 participants	14.8
	1,000 to 2,999 participants	14.1
	Up to 999 participants	32.8
	Not reported	22.7
Main type of participants	General public	45.3
	Students, young people	39.1
	Teachers, educators	4.7
	Media, journalists	1.6
	Other (e.g., elderly, parents, or scientists)	4.8
	Not reported	4.7
Feedback of participants	Positive feedback	68.0
	Mixed feedback	5.0
	Negative feedback	0
	Not reported	27.0
INDIRECT OUTCOMES		
Effects on participants^a^	Cognitive effects (e.g., interest)	46.9
	Emotional effects (e.g., enthusiasm)	22.7
	Attitudinal effects (e.g., attitude)	13.3
	Behavioral effects (e.g., intention)	15.6
	Not reported	42.2

a Multiple answers were possible.

Relatively few projects analyzed peoples’ initial response to social media or online content: 21.9% reported on social media engagement, focusing on the number of likes, shares, or uploads (e.g., in citizen science app). However, only 2.4% analyzed user comments to determine social media sentiments. The number of new fans, followers, or subscribers was reported by 12.5%. Again, such results were reported using different metrics and time periods, such as absolute follower growth versus relative follower growth, or followers per platform or aggregated across platforms. Therefore, the data presented were not comparable.

For *indirect outcomes*, just over half of the projects (57.8%) reported whether the activities had an effect on participants (see Table 4). Most projects reported that they had affected participants’ cognitions (46.9%), such as increased interest in the topic or science. Fewer projects indicated that the activity resulted in emotional effects (22.7%), such as that participants were enthusiastic or emotionally touched by the activity. Even fewer mentioned that their activities had resulted in attitudinal effects (13.3%), such as changes in participants’ attitudes toward climate change or disciplines such as chemistry. Slightly more reported on behavioral effects (15.6%) on audiences, such as the intention to study a STEM subject, to live more sustainably, or to visit the exhibition again. In 42.2% of projects, it remained unclear whether the science communication activity had affected participants. Overall, the analysis revealed a gap between the statements in the final reports and the grant applications, in which academics had promised more outcome-related effects, for example, on participants’ interest, knowledge, or intentions, than were eventually realized. A quarter provided supporting empirical evidence for the postulated effects on audiences (e.g., by referencing survey data or knowledge tests), while others remained rather vague or anecdotal (21.9%) (e.g., stating that teachers or parents indicated that the children had learned something) or offered no evidence at all (11.7%). Interestingly, a few projects also reported unintended negative effects: For example, some activities triggered discomfort (e.g., for the senses), amplified gender gaps, evoked inappropriate or hateful comments on social media, or led to heated debates about science, spirituality, or politics.

#### Impacts

Finally, the results show that long-term, substantial impact is rarely reported, and when it is, such narrative statements focus more on *scientific impact* than *societal impact*. 15.6% of projects reported on impact, meaning that the majority (84.4%) did not reflect on the broader value of the project. A discrepancy between expected and realized impact became evident, as nearly one-third of project applications had promised a long-term impact. Of the 15.6% of projects that reported an impact, most mentioned a scientific impact (7.8%), for example, through a collection of citizen science data or lasting changes in the way (early career) academics understand public outreach and engagement. Next were educational impacts (3.1%), for example, through the introduction of new teaching materials into school curricula. Furthermore, a few projects reported societal impacts on the Swiss public (2.3%), for example, in local communities where the activities took place. A few projects (2.4%) postulated economic impacts, for example, on the start-up scene or local economy, or environmental impacts, by contributing to sustained changes in climate-friendly behavior. Other types included policy impacts or institutional impacts (1.6%). In addition to narrative impact statements, academics reported traditional quantitative indicators of *scientific* impact in the standardized form provided by the SNSF: In absolute terms, the 128 projects resulted in 112 scientific publications, 17 awards, and 42 newly acquired third-party grants. In relative terms, 30% of projects resulted in publications, almost 10% received award(s), and 25.8% resulted in one or more third-party-funded follow-up projects.

## Discussion

The analysis of 128 science communication projects carried out by academics between 2012 and 2022 provides valuable insights into the evaluation practices and the outputs, outcomes, and impacts achieved by such projects.

First, the results show that most projects were evaluated (RQ1), but in one-third of projects, no evaluation was carried out by the academics. The study points to a paucity of more robust evaluation practices: The findings suggest that evaluations are hardly guided by logic models and underlying assumptions from the theory of change of how planned science communication activities lead to certain, logically anticipated outcomes that then lead to certain impacts. The projects often capture participants’ self-reported shifts in knowledge or attitudes, using summative evaluations that rely on cross-sectional data, rather than pre- and post-test-designs (e.g., knowledge tests). In line with previous research (Fu et al., 2016; Metcalfe et al., 2012), feedback forms or standardized surveys dominate, and only a quarter of projects combine different evaluation methods—although this would be necessary to track results along different stages. Overall, the analysis confirms previous studies which reveal that the evaluation of science communication is still fairly immature (e.g., Jensen, 2014; Metcalfe et al., 2012; Pellegrini, 2021; Sörensen et al., 2024; Ziegler et al., 2021).

Second, the analysis highlights the emphasis on demonstrating secondary outputs and direct outcomes (RQ2), that is, media coverage (75.8%), participant count (77.3%), and immediate feedback (72.0%). While nearly half of the projects reported changes in participants’ cognitions (46.9%), other indirect outcomes such as effects on attitudes, emotions, or behaviors, as well as long-term impact on society were often overlooked in reporting. Again, these results underline earlier research that shows a dominance of relatively simple output and outcome measurements in science communication (e.g., Bühler et al., 2007; Höhn, 2011; Sörensen et al., 2024; Weitkamp, 2015). 42.2% of projects did not mention any indirect outcomes for target groups and twice as many projects were silent on long-term impact, pointing to a more general lack of transparent reporting that has been problematized in previous research (Fu et al., 2016). Aside from the gap in omitting certain results, such as online reach (missing in 49.2% of projects) or online engagement (missing in 41.4% of projects), the projects that did track such outcomes often used different metrics to signal success. This renders evaluation outcomes incomparable and hinders a holistic understanding of science communication effectiveness on digital platforms.

There could be several reasons why there was a lack of sound evaluation: One explanation is that academics preferred investing time or budget in outreach activities rather than devoting resources to rigorous evaluations of one-off, temporary projects. Another plausible assumption is that academics lacked know-how of evaluation models and methods, or interest in self-evaluation (Ho et al., 2020; Metcalfe et al., 2012). Perhaps, communication experts involved in the projects provided too little advice on designing robust evaluations—or they themselves lacked the necessary time, resources, or methodological expertise (Jensen, 2014). Further reasons could also lie with the target audiences, who may have been reluctant to participate in surveys, as problematized in several reports, underlining the need for more unobtrusive and informal evaluation methods such as feedback walls (e.g., Grand & Sardo, 2017).

The fact that final project reports focused more on reporting outputs and direct outcomes might be related to the easy access to such (mostly digital) data (Volk & Buhmann, 2023) or because the funder specifically requested data on the volume of media output. An additional explanation could lie in project goals, which emphasized providing access to or raising awareness of scientific topics—and therefore required output-related metrics such as reach, visits, or likes—rather than outcome-related goals such as changing attitudes toward certain scientific topics. This focus on one-way knowledge transfer could also possibly reflect a deficit model understanding of science communication (Besley & Nisbet, 2013). Notably, in terms of indirect outcomes and impacts, the comparison of grant applications and final reports unravels that the results achieved often fall short of the promises made at the application stage. Given a trend toward inflationary impact statements driven by an increasingly fierce competition for funding (Chubb & Watermeyer, 2017), it was anticipated that grant applications would attempt to portray a project as potentially impactful. Indeed, grant applications appear to have over-promised effects and impacts. This may have been intentional to improve the chances of success in attracting the grant (King et al., 2015). However, it is also plausible to assume that academics were overly optimistic in their goal setting because they lacked training and experience with science communication projects (Ho et al., 2020; Phillips et al., 2018; Rodgers et al., 2018). The formulation of unrealistic goals might also be related to a systemic lack of a larger evidence base about what kind of changes can realistically be achieved through (often short- or one-time) exposures to science communication, particularly given that scientific topics are inherently complex and attitudes toward science are quite stable (Metag, 2017). In addition, the fact that communication effects are often indirect, conditional, person-specific or delayed can serve as an explanation for why some projects may not have found the direct effects expected.

In line with expectations, the projects were presented as successful in almost all final reports. Yet, the claims of success (e.g., positive feedback and changed attitudes) were rarely backed up with empirical evidence from evaluations, making them not particularly compelling and credible. Since the hoped-for effects and impacts did not always materialize to the desired extent, one might expect the grantees to justify why this was the case. A quarter to a third of the projects reflected on unrealized activities or target groups. In the remaining reports, the discrepancy between planned and achieved goals was not explained. Such a lack of transparency and full disclosure of project results (e.g., omitting the visitor count) can possibly be interpreted as a strategy to gloss over unachieved project goals or sweep unfulfilled promises under the table. In some cases, the impression was even created that some grantees made little effort in demonstrating accountability in the final reports. Particularly surprising is the fact that the societal impact achieved was hardly “marketized” and most final reports were silent on the subject. One explanation for this could be that statements about long-term societal impact naturally clash with the timing of reporting, as such impact may only not surface until months or years after the end of a project (Weitkamp, 2015). The emphasis on reporting scientific impact, in turn, may be due to the fact that academics are accustomed to signaling success in scientific currencies in final reports to funders (Brenninkmeijer, 2022).

## Conclusion

This study builds on a unique dataset of all 128 science communication projects funded by the SNSF from 2012 to 2022—presenting the first analysis of its kind in science communication research. The standardized content analysis of grant applications, final reports, and project data reveals that academics have developed a breadth of different outreach activities to inform, educate, or engage the public in science, which are often participatory, interactive, and dialogical in nature. However, a systematic assessment of the effectiveness of these activities is rare, as few projects apply rigorous evaluation designs and combine multiple evaluation methods. Furthermore, many projects emphasize media attention and participant count, but neglect reporting on the effects on audiences and societal impact.

This is also noteworthy in view of the limitations inherent in the dataset already discussed: Like many studies in the field of evaluation (e.g., Fu et al., 2016), the data sources analyzed are based on self-reports and not on independent external assessments. Very likely, academics had an interest in presenting their projects as “success stories” to the funding body (Jensen et al., 2022). Indeed, the majority of projects were described as successful, but evidence for the claimed success was often vague or missing. Future studies could use in-depth interviews or surveys of academics and funders to explore (lack of) motivation, competencies, and perceived challenges related to self-evaluation and impact assessment. Particularly, studies should further illuminate the pressures on academics to over-sell societal impact and analyze impact statements from a critical perspective (Chubb & Watermeyer, 2017; King et al., 2015).

To accelerate progress and leverage the effectiveness of science communication, a scientifically rigorous evaluation of science communication, including outputs, outcomes, and impacts, is essential. This study has made both a conceptual and an empirical contribution in this regard: The conceptual model presented in Figure 1 can inform future research and provide guidance for academics and practitioners in developing logic models for science communication evaluation, which describe the intended outcomes and impacts and define the logical pathways to achieve them. A recourse to the theory of change and communication effects research can help systematize different effects of science communication in a causal logical relationship and justify how certain science communication activities ultimately lead to societal impact. Future measurements need to consider the unique characteristics of science communication—especially the challenge of validly measuring change at the outcome stage—and address these with adequate pre- and post-test-designs. The study’s empirical evidence can stimulate reflection on evaluation requirements but also on realistic expectations about the effects of science communication in the context of short-term exposure, which is crucial as unfounded expectations can lead to disappointments both for academics, funders, and policy makers (King et al., 2015). Furthermore, the findings raise the question of the usefulness of academics’ self-evaluations, which may run the risk of becoming success stories rather than transparent reflections of project results and failures. This complicates meta-evaluations of evaluations and conclusions on the societal impact of funding schemes.

Importantly, the responsibility for better science communication evaluation should not be primarily assigned to academics (Palmer & Schibeci, 2014). Rather, greater collaboration should be incentivized between academics and professional science communication, who can advise researchers regarding the strategic use of media relations and social media (Besley et al., 2021) and take responsibility for the (quasi-external) evaluation of project results. At the same time, training of academics in science communication and its evaluation would still be desirable (Ho et al., 2020). Such training should also be offered by funding organizations (King et al., 2015): Several funders have developed toolboxes or guidelines for evaluating public engagement—such as the UK Research and Innovation (2020) in England, the SFI (2015) in Ireland, or CSIRO (2020) in Australia—including possible indicators, customizable evaluations templates (e.g., survey and feedback form), or best practice evaluations from previous projects. However, to make evaluation data comparable, clear guidelines and standards, or even selected mandatory success metrics, are needed for reporting results. Moreover, new approaches to impact assessment are needed that account for the long-term nature of impact and disciplinary differences (Brenninkmeijer, 2022). Funders need to address these issues if they require academics to conduct evaluations and intend to draw insights from such evaluation data (Fu et al., 2016; Jensen & Gerber, 2020), and they should also make these data available for meta-analytical research purposes. Addressing the identified challenges can contribute to a more robust evaluation of science communication, enhancing the potential for impactful and evidence-based science communication in the future.

## Supplemental Material

sj-docx-1-scx-10.1177_10755470241253858 – Supplemental material for Assessing the Outputs, Outcomes, and Impacts of Science Communication: A Quantitative Content Analysis of 128 Science Communication Projects Supplemental material, sj-docx-1-scx-10.1177_10755470241253858 for Assessing the Outputs, Outcomes, and Impacts of Science Communication: A Quantitative Content Analysis of 128 Science Communication Projects by Sophia Charlotte Volk in Science Communication

## Notes

1. A few projects made use of appendices, attaching, for example, the brochures or flyers created, a publication, or a media clippings report collected by an external service provider.

2. The form could be updated after project completion.

3. This was the case for activities such as a MOOC or virtual workshops during the COVID-19 pandemic, in which the number of registered online users or workshop participants was tracked.

4. Such publications used data collected through, for example, citizen science apps or virtual reality glasses.

5. Projects from the humanities received significantly more media attention (*M* = 17.5, MDN = 9) than projects from the life sciences (*M* = 6.7, MDN = 2.5) according to a Kruskal–Wallis test (*H* = 17.256, *p* = .004) with Dunn–Bonferroni correction (*z* = 3.025, *p* = .037); the effect was weak (*r* = .27). Exhibitions in museums, botanical gardens, or public places generated significantly more media coverage (*M* = 17.6, MDN = 11) than workshops or lectures (*M* = 2.8, MDN = 0), according to a Kruskal-Wallis test (*H* = 41.605, *p* = .002) with Dunn-Bonferroni correction (*z* = 4.884, *p* < .001); the effect was moderate (*r* = .43).

6. Some reported on unique visitors, others on returning visitors or visits; some counted per post, others per month, and still others aggregated all numbers by the end of the project, or aggregated numbers across all online platforms.

7. Exhibitions naturally had the highest attendance (*M* = 11,402, MDN = 5,000; *SD* = 15,110) and were significantly different from projects that created learning or teaching material for schools (*M* = 250, MDN = 170, *SD* = 23) according to a Kruskal-Wallis test (*H* = 35.549, *p* = .012) with Dunn-Bonferroni correction (*z* = 4.649, *p* < .001); the effect was moderate (*r* = .47). However, the latter projects often had multiplier effects, for example, when the participant group consisted of teachers who were trained to use teaching materials—an effect that is difficult to measure.
