# Supplementary material for: Assessing the Outputs, Outcomes, and Impacts of Science Communication: A Quantitative Content Analysis of 128 Science Communication Projects
Source: Sci Commun. 2024 Jun 17;46(6):758–89. doi: 10.1177/10755470241253858 (PMC11496030; doi:10.1177/10755470241253858)
Supplement: sj-docx-1-scx-10.1177_10755470241253858 – Supplemental material for Assessing the Outputs, Outcomes, and Impacts of Science Communication: A Quantitative Content Analysis of 128 Science Communication Projects [file sj-docx-1-scx-10.1177_10755470241253858.docx]

**Supplementary Material**

**Assessing the outputs, outcomes and impacts of science communication:**

**A quantitative content analysis of 128 science communication projects**

Overview

[SM1. Sample description 2](#_Toc155186091)

[SM2. Data protection and confidentiality 4](#_Toc155186092)

[SM3. Descriptive data 5](#_Toc155186093)

# **SM1. Sample description**

The 128 sampled science communication projects received an average of 158’168 CHF funding from the SNSF and lasted 25.9 months (see Table 1). 49% of the projects received additional third-party funding, on average 50’068 CHF, but amounts varied greatly (*SD* = 102’764 CHF). The average amount of total funding per project was 208’447 CHF.

*Table 1*. Sample description (N = 128 projects)

| **Variable** | **Operationalization** | **Range** | **M** | **Median** | ***SD*** |
| --- | --- | --- | --- | --- | --- |
| Funding | Total amount of funding granted by SNSF and third parties in Swiss Francs (CHF) | 17’280-792’131 | 208’447 | 190’914 | 128’079 |
| Duration | Project duration in months | 4-62 | 25.9 | 24.0 | 10.82 |
| Project partners | Aggregated number of project partners involved  0 = None/not reported  1 = 1-3 partners  2 = 4-10 partners  3 = 11 or more partners | 0-3 | 2.28 | 2.00 | .793 |

The projects collaborated with a range of partners: 48% had more than eleven collaborators, 34% worked with up to ten partners, and 16% worked with up to three collaborators; only 2% did not list any partner. Collaborators most often came from other scientific institutions or associations (84%), the creative field (71%; e.g., artists, filmmakers), or (non-academic) professional networks (64%; e.g., teachers’ association). Moreover, the majority of projects could also build on the expertise of a communication professional (87%), e.g., through the official support of the university’s communication department.

Seven out of ten applicants were male (see Table 2); the average age was 54.5 years (*SD* = 8.6). More than two-thirds held a position of professor, while a fourth were established researchers with a Ph.D. 67.2% worked at a research university, while fewer worked at a university of applied science or technical university (each 10.9%). Researchers came from various scientific disciplines, led by the natural/life sciences (36.0%) and the humanities (35.2%), followed by the social sciences (14.8%).

*Table 2*. Description of project leaders (N = 128 projects)

| **Variable** | **%** |
| --- | --- |
| **Gender** |  |
| Male | 70.3 |
| Female | 29.7 |
| **Position** |  |
| Professor | 68.8 |
| PhD/Dr. | 24.2 |
| MA/MSc | 3.9 |
| other/not mentioned | 3.2 |
| **Institution** |  |
| Research University | 67.2 |
| University of Applied Sciences | 10.9 |
| Technical University | 10.9 |
| University of Teacher Education | 3.9 |
| Other | 7 |
| **Discipline** |  |
| Humanities | 35.2 |
| Social Sciences | 18.8 |
| Life Sciences | 17.2 |
| Mathematics, Natural Sciences | 14.8 |
| Engineering Sciences | 9.4 |
| Medicine | 4.7 |

# **SM2. Data protection and confidentiality**

For each project, three sources of data were obtained from the archive of the SNSF about all 128 science communication projects that were successfully funded by the end of May 2022:

1. *grant applications* (in PDF format), in which grant applicants described the planned project goals, target audiences, outreach activities and communication plan, evaluation plan, and envisioned outputs, outcomes, and impacts,
2. *final project reports* (in PDF format), in which grantees described the realized project goals, target audiences, outreach activities and communication measures, evaluation conducted, and the achieved outputs, outcomes, and impacts, and
3. *project data* (in Excel format), which contained standardized project information partly collected by the SNSF (e.g., amount of funding, duration) and selected self-reported project results from a standardized entry form (e.g., achieved media coverage, achieved scientific impacts) that grantees filled in upon (and even after) project closure.

The first two sources—grant applications and final project reports—partly contained non-anonymized data (e.g., the name of the applicant’s institution, partner institutions), while the third data source was provided by SNSF in a form containing no personal information or data. As part of the contract with the SNSF, the author and the three coders signed the Swiss Data Flow Agreement (in particular Article 22 paragraph 1 of the Swiss Federal Act on Data Protection, FADP), which regulates the confidentiality of personal data. During the coding process, no confidential institutional or personal information data were recorded from the grant applications and final reports. Therefore, the final dataset no longer contained any sensitive information, and all data were analyzed in anonymized form so that no person- or project-specific analyses were possible, i.e., individual grantees or projects could not be identified. At the end of the contract term with the SNSF, the original data sources obtained by the author were destroyed in accordance with the Swiss Data Flow Agreement. While the University’s ethics committee did not require ethics approval for this study, the author adhered to APA ethical standards throughout the research.

# **SM3. Descriptive data**

Table 3 shows descriptive data for ordinal and metric variables at the output and outcome level.

*Table 3*. Descriptive data for selected variables (N = 128 projects)

| **Stage** | **Variable** | **Operationalization** | **Range** | **M** | **Median** | ***SD*** |
| --- | --- | --- | --- | --- | --- | --- |
| **PRIMARY OUTPUTS** | | |  |  |  |  |
| **ACTIVITIES** | |  |  |  |  |  |
|  | Number of activities | Aggregated number of possible outreach activities (e.g., installation, guided tour, workshop) | 1-9 | 4.44 | 4.33 | 1.990 |
| **COMMUNICATION** | | | | | | |
|  | Number of online channels | Aggregated number of possible online channels (website, Facebook, Twitter other/unspecified)  0 = Not reported | 0-5 | 1.74 | 1.57 | 1.247 |
|  | Number of marketing measures | Aggregated number of possible marketing measures (e.g., newsletter, media relations, advertisement)  0 = Not reported | 0-5 | 1.96 | 1.94 | 1.499 |
| **SECONDARY OUTPUTS** | | |  |  |  |  |
|  | Media coverage | Aggregated number of the media reports about the project that appeared on TV, radio, print / online news, and other online media (e.g., podcast, blogs)*  0 = Not reported | 0-80 | 10.5 | 4.7 | 15.68 |
| **DIRECT OUTCOMES** | | | | | | |
|  | Number of participants | Aggregated number of all participants in a project’s activities** | 48-55’278 | 6’438 | 1’395 | 11’233 |
| **SCIENTIFIC IMPACTS** | | |  |  |  |  |
|  | Publication | Aggregated number of publications  0 = Not reported | 0-32 | .88 | 0 | 3.087 |
|  | Award | Aggregated number of awards  0 = Not reported | 0-3 | .13 | 0 | .441 |
|  | Follow-up project | Aggregated number of follow-up projects  0 = Not reported | 0-3 | .33 | 0 | .629 |

*Note:* *N = 127 (excluding one extreme case), **N = 98 cases (excluding cases with missing values and one extreme case)
